# Spectrum of germline cancer susceptibility gene mutations in Turkish colorectal cancer patients: a single center study

**DOI:** 10.3906/sag-2002-46

**Published:** 2020-06-23

**Authors:** Haktan Bağış ERDEM, Taha BAHSİ

**Affiliations:** 1 Department of Medical Genetics, University of Health Sciences, Dr. Abdurrahman Yurtaslan AnkaraOncology Training and Research Hospital, Ankara Turkey

**Keywords:** Colorectal cancer, genetics, hereditary cancer, next-generation sequencing

## Abstract

**Background/aim:**

Quarter of colorectal cancer patients have a family history and 6% of these comprise hereditary cancer syndromes. For developing national health strategies for genetic screening, it is crucial to determine the spectrum of damaging alterations in causative genes and to describe frequent founder mutations.

**Materials and methods:**

One hundred and thirty six unrelated colorectal cancer cases were investigated. Qiagen large hereditary cancer panel and Hereditary Cancer Solution v1.1 panel were used for sequencing. The sequencing process was performed on the Illumina MiSeq system. The data analyses were performed on QIAGEN Clinical Insight (QCI™) Analyze software and Sophia DDM software.

**Results:**

Of 136 patients, 11 (8%) were found to carry a pathogenic and 2 (1.4%) were found to carry a likely pathogenic mutation. Altogether, 12 different pathogenic and likely pathogenic mutations were detected.

**Conclusion:**

This study is the first study in Turkish colorectal cancer patients using next-generation sequencing. Point mutation screening in the families of patients with mutations will be able to identify individuals at risk in a cost-effective manner.

## 1. Introduction

Colorectal cancer (CRC) is the third most common cancer among both sexes and the fifth leading cause of cancer death in Turkey. According to the guidelines, 60% of CRC deaths could be prevented with screening. Approximately, 25% of people diagnosed with CRC have a family history and 6% of these comprise hereditary cancer syndromes for which deleterious genomic variations are found in cancer susceptibility genes. Therefore, the hereditary component of CRC becomes more important when compared to other cancers [1]. 

Hereditary CRC is divided into two groups as nonpolyposis syndromes and polyposis syndromes [2]. Hereditary nonpolyposis colorectal cancer (HNPCC) accounts for 83% of hereditary colorectal cancers, which is also known as Lynch syndrome. Lynch syndrome is inherited in an autosomal dominant pattern, caused by mutations in mismatch repair genes. Mainly mutations of MLH1, MSH2, PMS2, and MSH6 genes, which damage the mismatch repair mechanism, are responsible for the disease [3]. Microsatellite instability (MSI) analysis is an alternative for evaluating HNPCC at somatic level. High-level MSI (MSI-H) is a diagnostic marker for HNPCC. Moreover, somatic BRAF mutations and germline MLH1 hypermethylation have diagnostic value. An individual carrying a germline MLH1 or MSH2 mutation is estimated to have a cumulative lifetime cancer risk of up to 74% and 40% for CRC and endometrial cancer, respectively [4,5].

Development of multiple adenomatous polyps throughout the gastrointestinal tract is characterized for familial adenomatous polyposis (FAP). Without precaution, the risk of developing CRC in FAP patients by the age of 40 is 100%. Mutations of APC gene are responsible for FAP [6]. Prevalence of FAP is approximately 1 in 10,000 individuals and accounts for 0.5–1% of all CRC cases [7]. FAP can cause papillary thyroid cancer and hepatoblastoma as well as CRC.

MUTYH-associated polyposis (MAP), Peutz-Jeghers syndrome (PJS), juvenile polyposis (JPS), and Cowden syndrome are the other rare CRC predisposition syndromes. The clinical features of MAP are similar to that of FAP but while hundreds of thousands of polyps are seen in FAP, this number is between 5 and 100 in MAP. Mean age at diagnosis of MAP patients is also higher than FAP. Biallelic pathogenic variants of MUTYH gene can cause the disease [8]. PJS is characterized by benign hamartomatous polyps in the gastrointestinal tract and hyperpigmented macules on the lips and oral mucosa. The hamartomatous polyps in PJS are most commonly located in the small intestine but may also occur along jejunum and ileum. The risk of CRC, and breast, pancreatic, stomach, testical, ovarian, lung, and cervical cancer is increased in these patients. STK11 is the causative gene of PJS. Prevalence of PJS is estimated nearly 1 in 100,000 individuals [9]. JPS is a rare autosomal dominant disease, identified by the presence of hamartomatous polyps in the digestive tract and increased cancer risk of CRC, gastric, and pancreatic cancers. Disease-causing genes are SMAD4 and BMPR1A for this syndrome. JPS has a prevalence of approximately 1 in 100,000 [10]. Cowden syndrome is also characterized by multiple hamartomatous lesions, particularly in skin, gastrointestinal tract, breast, and thyroid gland. Breast and thyroid cancers are the most expected neoplasms for Cowden syndrome. PTEN gene mutations have been described in these groups of patients [11]. According to recent publications, germline pathogenic variants in POLD1, POLE, and GREM1 have been associated with CRC tendency [12,13].

Sanger sequencing of cancer susceptibility genes one by one was not widespread in routine diagnosis because it was time-consuming and not cost-effective. With the spread of next-generation sequencing (NGS)-based hereditary cancer panels, high-throughput sequencing was made possible, databases of different populations have been constructed, and the spectrum of cancer predisposition genes has been provided. In case of insufficient diagnostic ability of these panels, whole exome/genome sequencing and transcriptome studies have been brought to the agenda [14]. With the new genes discovered as a result of research, the content of hereditary cancer panels is expanding day by day.

The prevalence of cancer susceptibility gene mutations varies with ethnicity and region. There may be contradictory views about the pathogenic potential of the variants. In order to make a clear interpretation, it is important to provide genetic counseling in the light of detailed clinical information of the patient. Although data from different populations presented with databases and articles continues to increase day by day, the majority of studies include the prevalence and mutation spectrum cancer susceptibility genes in European, Asian, North American, African, and African–American populations [14,15]. Hereby, there is a need for better understanding the mutation spectrum of these genes and cancer risk prediction in Turkish people. For developing national health strategies for genetic screening, it is crucial to determine the spectrum of damaging alterations in causative genes and to describe frequent founder mutations.

## 2. Materials and methods

### 2.1. Patients and samples

A total of 136 subjects were included in the present study at University of Health Sciences, Dr. Abdurrahman Yurtaslan Ankara Oncology Training and Research Hospital, Medical Genetics Clinic, between 2017 and 2019. Ethical committee of Dr. Abdurrahman Yurtaslan Ankara Oncology Training and Research Hospital approved the study (2019-11/443). Written informed consent was obtained from all patients before testing for the use of their DNA samples for research purposes. Family histories were recorded for all the patients, including first-, second-, and third-degree relatives on both the maternal and paternal sides of the family, and covering at least three generations. Personal and clinical data (sex, age of onset, histopathologic characteristics, immunohistochemistry, and pathology results) were taken from an inspection of digital medical archive. All the patients were unrelated and provided genetic testing criteria in agreement with the National Comprehensive Cancer Network (NCCN) Guidelines for Genetic/Familial High-Risk Assessment: Colorectal.

### 2.2. DNA extraction

Blood samples were collected into EDTA tubes. Patients’ DNAs were extracted with QIAcube® automated DNA isolation system (Qiagen Inc. Mississauga, ON, Canada). Isolated DNA samples were stored at –20 °C. Before sequencing, the DNA concentration and quality were measured with NanoDrop (ND-1000) spectrophotometer (Nano-Drop Technologies, Wilmington, DE, USA) for OD260/OD280, 1.8 to 2.0.

### 2.3. Genetic testing

Qiagen large hereditary cancer panel (Qiagene, Hilden, Germany) and Hereditary Cancer Solution v1.1 panel (Sophia Genetics, Saint‐Sulp) were used for sequencing. The sequencing process was performed on the Illumina MiSeq system (Illumina Inc., San Diego, CA, USA). The data analyses were performed on QIAGEN Clinical Insight (QCI™) Analyze software (QIAGEN, Hilden, Germany) for Qiagen large hereditary cancer panel and Sophia DDM software (Sophia Genetics, Saint‐Sulp) for Hereditary Cancer Solution v1.1 panel. The gene content of these hereditary cancer panels was listed in Table 1. Sanger validation was performed for homopolymer regions, low quality variants, insertions and/or deletions, splice site alterations, and novel variants.

**Table 1:** Gene content of hereditary cancer panels.

Qiagen QiaseqHereditary Custom Cancer Panel	AIP, APC, ATM, ATR, AXIN2, BAP1, BARD1, BLM, BMPR1A, BRCA1, BRCA2, BRIP1, BUB1B, CDH1, CDK4, CDKN2A, CHEK2, CTNNA1, EPCAM, FAM175A, FANCC, FLCN, GALNT12, GEN1, GPC3, GREM1, HOXB13, MET, MLH1, MRE11A, MSH2, MSH6, MUTYH, NBN, NTHL1, PALB2, PALLD, PIK3CA, PMS1, PMS2, POLD1, PRSS1, PTCH1, PTEN, RAD50, RAD51B, RAD51C, RAD51D, RET, RINT1, SDHB, SDHC, SDHD, SMAD4, SMARCA4, STK11, TP53, VHL, XRCC2
Sophia Hereditary Cancer Solution Panel	ATM, APC, BARD1, BRCA1, BRCA2, BRIP1, CDH1, CHEK2, EPCAM, FAM175A, MLH1, MRE11A, MSH2, MSH6, MUTYH, NBN, PALB2, PIK3CA, PMS2, PMS2CL, PTEN, RAD50, RAD51C, RAD51D, STK11, TP53, XRCC2

### 2.4. Variant classification

The recent ACMG/AMP guideline for standardized variant interpretation in Mendelian disorders was used for classification. Pathogenic variants are well-established disease- causing DNA changes in in-house database and/or literature. The main evaluation criteria are represented by strong clinical findings and family history, independent confirmatory observations, and supporting pathogenicity functional studies. Likely pathogenic variants are considered the probable cause of the disease, or the effect on the protein function is predicted to be likely deleterious (>90% probability to cause the disease). Variant of uncertain significance (VUS) alterations are genetic variants with unknown or questionable impact on the disease. These variants are typically very rare and predicted to be deleterious.

## 3. Results

Of the 136 patients, 11 (8%) were found to carry a pathogenic and 2 (1.4%) were found to carry a likely pathogenic mutation. Altogether, 12 different pathogenic and likely pathogenic mutations were detected. The pathogenic and likely pathogenic variations were located in ATM, BRCA2, CHEK2, MLH1, MSH2, MUTYH, PMS2, RINT1, and TP53 genes. MUTYH:c.884C>T (NM_001128425) pathogenic variation is the only pathogenic/likely pathogenic variant, described in more than one patient. One patient was biallelic, and the other patient was monoallelic with another pathogenic variant, MUTYH:c.536A>G, at transposition. This compound heterozygous patient was also the only pathogenic compound heterozygous patient of this study (Table 2). 

**Table 2:** Described pathogenic and likely pathogenic variants in CRC patients.

Gene	Transcript	cDNA change	Protein change	dbSNP	Consequence	Variant type	n
ATM	NM_000051	c.7788G>A	p.Glu2596=	rs587780639	Synonymous	Pathogenic	1
BRCA2	NM_000059	c.9317G>A	p.Trp3106Ter	rs80359205	Nonsense	Pathogenic	1
CHEK2	NM_007194	c.1260C>A	p.Cys463Ter	rs762205611	Nonsense	Pathogenic	1
MLH1	NM_000249	c.1609C>T	p.Gln537*	rs63751277	Nonsense	Pathogenic	1
MSH2	NM_000251	c.2362dupA	p.Thr788Asnfs*11	rs63750463	Frameshift	Pathogenic	1
MUTYH	NM_001128425	c.536A>G	p.Tyr179Cys	rs34612342	Missense	Pathogenic	1
MUTYH	NM_001128425	c.545G>A	p.Arg182His	rs143353451	Missense	Pathogenic	1
MUTYH	NM_001128425	c.884C>T	p.Pro295Leu	rs374950566	Missense	Pathogenic	2
MUTYH	NM_001128425	c.14734_1439delGGA	p.Glu480del	-	In frame	L.Pathogenic	1
PMS2	NM_000535	c.690_691delGT	p.Phe231Trpfs*17	rs1064795447	Frameshift	Pathogenic	1
RINT1	NM_021930	c.1333+1G>A	-	rs375350359	Splice defect	L.Pathogenic	1
TP53	NM_001276696	c.37C>T	p.Gln13Ter	-	Nonsense	Pathogenic	1

L.Pathogenic: likely pathogenic

VUS alteration was detected in 40 patients (29.4%) with 38 different variants (Table 3). The spectrum of pathogenic and likely pathogenic mutations comprises 2 (15.3%) frame-shift variants, 4 (30.7%) nonsense variants, 4 (30.7%) missense variants, 1 (7.7%) splice site defect, 1 (7.7%) inframe variant, and 1 (7.7%) synonymous variant (Table 2). The spectrum of VUS variants comprises 37 (92.5%) missense variants and 3 (7.5%) splice site alteration (Table 3). All the pathogenic, likely pathogenic, and VUS variants are listed in Tables 2 and 3.

**Table 3:** Described VUS variants in CRC patients.

Gene	Transcript	cDNA change	dbSNP	Consequence	n
APC	NM_000038	c.2438A>G	rs201522866	Missense	1
APC	NM_000038	c.3920T>A	rs1801155	Missense	2
APC	NM_000038	c.5609A>G	rs1189738231	Missense	1
ATM	NM_000051	c.2021A>G	rs201762714	Missense	1
ATM	NM_000051	c.6869A>C	-	Missense	1
ATM	NM_000051	c.7082T>C	rs1169558907	Missense	1
ATM	NM_000051	c.3402+16A>G	rs63382531	-	1
BARD1	NM_000465	c.586A>G	rs376259263	Missense	1
BLM	NM_000057	c.11T>C	rs144706057	Missense	1
BRCA1	NM_7294	c.3448C>T	rs80357272	Missense	1
BRCA1	NM_7294	c.4342A>G	rs80357486	Missense	1
BRCA2	NM_000059	c.5070A>C	rs56087561	Missense	1
BRCA2	NM_000059	c.5092T>C	-	Missense	1
BRCA2	NM_000059	c.5495C>A	rs138489917	Missense	1
BRIP1	NM_032043	c.1255C>T	rs150624408	Missense	1
BRIP1	NM_032043	c.3178G>A	rs149016505	Missense	1
BUB1B	NM_001211	c.522A>G	-	Missense	1
CDH1	NM_004360	c.184G>A	rs587781898	Missense	1
CDH1	NM_004360	c.2387G>A	rs587782549	Missense	1
CDH1	NM_004360	c.2359G>A	rs766270336	Missense	1
CDH1	NM_004360	c.2595G>C	rs778019174	Missense	1
CHEK2	NM_007194	c.944G>A	-	Missense	1
CHEK2	NM_007194	c.1556G>T	rs587780180	Missense	1
EPCAM	NM_002354	c.28G>C	rs863224709	Missense	1
MLH1	NM_000249	c.1876T>C	rs377241633	Missense	1
MRE11A	NM_005591	c.818C>G	rs143400546	Missense	1
MSH2	NM_000251	c.-107C>A	rs587782649	-	1
MSH2	NM_000251	c.435T>G	rs63750124	Missense	2
MSH2	NM_000251	c.2606C>A	rs730881772	Missense	1
MUTYH	NM_001128425	c.821G>A	rs149866955	Missense	1
PALB2	NM_024675	c.3201+4delA	-	-	1
PMS1	NM_000534	c.2722T>A	-	Missense	1
PMS2	NM_000535	c.2392T>C	-	Missense	1
POLD1	NM_001256849	c.455C>T	rs41563714	Missense	1
POLD1	NM_001256849	c.2293G>A	rs759190487	Missense	1
RAD50	NM_005732	c.2651G>A	rs558302979	Missense	1
RET	NM_020975	c.224C>T	rs142641173	Missense	1
RET	NM_020975	c.628G>A	rs1060500762	Missense	1

## 4. Discussion

With the expansion of precision medicine, analysis of cancer-related genes at both germline and somatic levels will become even more important in diagnosis, susceptibility, prognosis, treatment resistance, and recurrence assessments. In this context, Dr. Abdurrahman Yurtaslan Ankara Oncology Training and Research Hospital is the only third-step, national oncology hospital, which is affiliated with Turkish Ministry of Health. Since 2017, germline hereditary cancer panel tests have been performed in our molecular genetics laboratory within the scope of public health services.

With four pathogenic, one likely pathogenic, and one VUS changes, the most reported gene in our study was MUTYH, but as it was mentioned before, only the biallelic damaging variants can cause MAP (Tables 2 and 3). Among these six samples only MUTYH:c.884C>T (NM_001128425) variant was biallelic (homozygous for one patient and compound heterozygous for one patient). Previously, this variant was observed in both the homozygous and compound heterozygous state in individuals affected with MAP [16,17]. Two pathogenic mutation carriers (MUTYH:c.536A>G (NM_001128425), MUTYH:c.545G>A (NM_001128425)) and one likely pathogenic mutation carrier (MUTYH:c.1437_1439delGGA) had not sufficient evidence for explaining the molecular etiology of MAP. Copy number variations (CNVs) can complete the second hit. Although the bioinformatics pipelines allow us to evaluate the CNVs, the gold standard genetic test for detecting CNVs is multiplex ligation-dependent probe amplification analysis (MLPA). Until now, a gross (>4.2 kilobase) deletion covering exons 4-16 has been described in three MAP patients from Spain, France, and Brazil, indicating a possible southern European founder variant [18–20]. An exon 15 deletion has also been reported recently [21]. Therefore, MLPA should be performed for excluding second hit missing for MUTYH gene in these samples, but MLPA for MUTYH gene was not available for this study. In different context, there are also studies about the effect of monoallelic MUTYH mutations on CRC risk. Although not as high penetrance as in biallelic cases, the increased risk of CRC has also been reported in MUTYH mutations in monoallelic cases [22]. Pedigrees of the cases of this study also support this thesis. Further investigation is needed for exploring the risk levels between biallelic and monoallelic cases to give an effective genetic counseling to affected individuals.

Among the syndromes causing CRC, the most common one is Lynch syndrome (HNPCC) in the patients included in this study. CHEK2:c.1260C>A (NM_007194), MLH1:c.1609C>T (NM_000249), MSH2:c.2362dupA (NM_000251), PMS2:c.690_691delGT (NM_000535) are detected pathogenic variants with HNPCC patients (Table 2). Although CHEK2 is not a mismatch repair gene (MMR), mutations of this gene could be responsible for some cases of Lynch syndrome [23]. The proband, which carries CHEK2 mutation, has overlapping phenotype with HNPCC, and the variation meets the pathogenicity criteria as well. Main genes causing Lynch syndrome are MSH2 (50%), MLH1 (30–40%), MSH6 (7–10%), PMS2 (<5%), and EPCAM (1-3%) respectively [24]. This study is the first study in Turkish Lynch syndrome patients using next-generation sequencing technique. For this reason, there is no study to be referenced about the frequency of Lynch syndrome mutations in Turkish population. The frequency of mutations detected among CRCs is relatively different from the literature, although the sample is not sufficient to accurately assess this, suggesting that the distribution of mutations in different populations may also vary. The mutation spectrum of Lynch syndrome can be revealed with the consortiums that will be formed by the collaboration of the centers having data belonging to the Turkish population. An additional contribution of such databases would be the healthier reporting of variants reported as VUS due to lack of in-house data.

The most important cancer type related with BRCA1/2 is breast and ovarian cancer. Other solid tumor cancers commonly seen in BRCA1/2 carriers are pancreas and prostate tumors [25]. Although the previous view suggests that only the BRCA1 gene increases CRC risk, recent studies suggest that both the BRCA1 and BRCA2 genes may slightly increase CRC risk [26]. The proband of this study, with pathogenic BRCA2:c.9317G>A (NM_000059) variant, was male and support this evidence with the phenotype and family history. There are two more male CRC cases in third-degree relatives and there are several breast cancer cases among both sexes on the pedigree.

Li-Fraumeni syndrome is associated with developing of several types of cancer, and the germline TP53 mutations are responsible for the phenotype. Pathogenic TP53:c.37C>T (NM_001276696) mutation detected in CRC case was also compatible with Li-Fraumeni syndrome [27]. ATM is altered in 5.58% of all cancers and CRC is one of the important cancer types in these. The detected variant, ATM:c.7788G>A (NM_000051), has synonymous effect and does not cause any amino acid alteration on the protein. While biallelic mutations of ATM cause ataxia telangiectasia syndrome, monoallelic mutations predispose cancer. This variant was described with both ataxia telangiectasia syndrome and hereditary cancer [28]. Splice site alteration was detected at in silico splice site analysis tool, Human Splicing Finder http://www.umd.be/HSF/. Although the association of RINT1 gene with breast cancer has been shown, there is no detailed study of its effect. It has been previously reported that RINT1 may be associated with Lynch syndrome [29]. A novel splice variant RINT1:c.1333+1G>A (NM_021930) was detected in a CRC patient. Although this variant was not seen in the healthy population, it was found that the splice region was affected according to Human Splicing Finder. This novel variant detected in the study is thought to contribute to the effect of RINT1 mutations on CRC.

In our study, APC gene mutation was not detected in patients who underwent hereditary cancer panel. Due to the fact that FAP has distinctive clinical features, single gene analysis is performed instead of large panels in terms of cost effectiveness in routine genetic diagnosis. Since hereditary cancer panels are commercially produced kits, the patients included in the study were automatically evaluated for APC gene. Therefore, our study is not informative in terms of the proportion of FAP patients in CRC patients and the mutation distribution of the APC gene.

Hereditary cancer panel application is becoming more common in patients presenting with a high number of CRCs or additional cancers in the family. Point mutation screening in the families of patients with mutations will be able to identify individuals at risk in a cost-effective manner. Further studies in Turkish population will contribute to the spectrum of germline mutations in CRC patients in the future.

## Conflict of interest

The authors declare that there is no conflict of interest.
